# Population structure and genome-wide association study of body conformation traits of two native goat breeds in China

**DOI:** 10.5713/ab.25.0334

**Published:** 2025-08-25

**Authors:** Rong Yang, Di Zhou, Yanli Lv, Xingzhou Tian, Liqun Ren, Fu Wang, Zhengang Guo, Yongju Zhao, Jipan Zhang

**Affiliations:** 1 Guizhou Provincial Breeding Livestock and Poultry Germplasm Determination Center, Guiyang, China; 2 College of Animal Science, Guizhou University, Guiyang, China; 3 College of Animal Science and Technology, Southwest University, Chongqing, China

**Keywords:** Body Conformation Trait, Genome-wide Association Study, Goat, Population Structure, Single Nucleotide Polymorphism

## Abstract

**Objective:**

Body conformation traits directly impact carcass performance in the meat goat industry. This study explored the population genetics of two Chinese goat breeds and identified the genomic variants associated with their body conformation traits.

**Methods:**

The Guizhou black goat (GBG, n = 104) and Hezhang black goat (HBG, n = 100) underwent genotyping through whole-genome sequencing and phenotyping by measuring their body height (BH), body length (BL), chest depth (CD), chest width (CW), chest girth (CG), rump width (RW), rump height (RH), and cannon circumference (CC).

**Results:**

The relatedness analysis showed that these goats exhibited low genetic kinship-related, with the GBG and HBG being relatively independent, albeit with some genetic introgression present. The lambda values showed that the reliability of the genome-wide association studies (GWAS) model, identifying a total of 33, 1, 6, 2, 5, 10, 21, and 13 single nucleotide polymorphisms (SNPs) as significantly correlated (p<8.33e-8) with BH, BL, CD, CW, CG, RW, RH, and CC, respectively. The GWAS for BH and RH identified the greatest number of significant SNPs, with a substantial overlap among them, mainly located in four regions: chr13_63286230-69784740 (10 SNPs), chr14_60354209-60376549 (six SNPs), and chr15_65605417-73873841 (five SNPs), and chr23_42819635-43332716 (nine SNPs). Individuals with a greater number of these SNPs displayed elevated BH and RH values. Following the annotation of all significant SNPs, 102 genes within a ±100 Kb region were identified. The most significantly enriched KEGG pathway was “Olfactory transduction”, while the most significantly enriched GO terms included “cellular process” and “molecular transducer activity”.

**Conclusion:**

This study investigated the population genetics of two prominent Chinese goat breeds and identified several SNPs that are significantly associated with body conformation traits. These findings offer biological insights into enhancing growth performance and hold significant potential for practical application in the genomic selection of meat goats.

## INTRODUCTION

Goats are valuable economic livestock in developing countries, especially in Asia and Africa, providing essential products such as milk, meat, mohair, cashmere, and fur [1]. China ranks first globally in both the number of goat breeds and the total population of goats raised [2]. More specifically, China has 69 native goat breeds listed in the National Catalogue for Livestock and Poultry Genetic Resources in 2024, where most are concentrated in the southern regions. There are two black goat breeds in the Guizhou province, namely the Guizhou black goat (GBG, [3]) and the Hezhang black goat (HBG), and both are characterized by a solid black coat and a relatively small but robust physique, which is well-adapted to the local hilly terrain and subtropical climate. Although it is believed that diverse natural and artificial selection pressures have shaped goat breeds that are well-adapted to local environments, improving goat body size to enhance farming benefits is a primary demand of the local livestock industry. Despite this, attention to these breeds remains limited.

An improvement in living standards in China has led to a rapid increase in the demand for goat products and also prompted the selection of goats with larger body sizes [4]. Body shape can, to a certain extent, reflect production benefits such as milk yield, cashmere or wool yield, and slaughter performance, and larger animals often produce more milk due to their larger size and greater amount of mammary gland tissue, giving them a higher production potential [5]. Similarly, bigger animals typically have a larger skin surface area, resulting in a larger effective area for cashmere or wool [6], and larger animals typically possess more muscle and fat, resulting in a higher carcass weight, net meat weight, and slaughter rate [7–9]. Therefore, breeding goats with a larger body size is more conducive to the efficient production of the meat goat industry. To this end, goat shape is determined by the following traits: body height (BH), body length (BL), chest depth (CD), chest width (CW), chest girth (CG), rump width (RW), rump height (RH), and cannon circumference (CC). Unfortunately, these traits are polygenic and highly environmentally influenced, making gene identification difficult [10]. Therefore, the pursuit of increased meat production underscores the critical need to uncover the associated genetic variants in goats, prompting the adoption of innovative methodologies, such as genome analysis.

Genome-wide association studies (GWAS) are widely used for studying the relationship between genetic variation and complex traits [11,12] and rely on linkage disequilibrium (LD) for discovering causative single nucleotide polymorphisms (SNPs) and candidate genes. Techniques for genotyping include micro-array, whole genome sequencing, and simplified genome sequencing, among others. Although whole genome resequencing is more costly, more efficient SNPs can be obtained, and based on the GWAS method, candidate genes associated with milk quality [13,14], animal fiber traits [15,16], meat quality [17], growth [18,19], and body conformation (such as body height, and body length) [20,21] have been uncovered in goats. The genetic revelations derived from these studies not only offer prospective trajectories for selective breeding programs but also establish a foundation for the sustainable augmentation of productive performance.

This study aimed to investigate the population structure and genomic variants associated with body conformation traits in GBG and HBG. Based on phenotypical data and whole genome sequences from 204 goats, a phenotypic analysis, relatedness analysis, population structure analysis, GWAS, and gene enrichment analysis were performed to identify genes associated with the body conformation traits in these goats.

## MATERIALS AND METHODS

### Animals and body conformation traits

This experiment was approved by the Animal Care and Use Committee of Southwest University (No. IACUC-20230417-02) and Guizhou University (No. EAE-GZU-2023-E047). Guizhou black goats (GBG, n = 104) and Hezhang black goats (HBG, n = 100) from Guizhou province, China, were investigated in Anshun and Bijie City, respectively. Adult goats were randomly selected from different villages and farms, and animals with direct kinship were avoided. Sample collection and trait determination were conducted by a team of four, where one person was responsible for keeping the goat calm, while another measured the goat’s body conformation traits. A third collected 3–5 mL of blood from the goat’s jugular vein, and the fourth recorded the data. The blood samples were immediately placed in an icebox and transported to the laboratory to be stored at −80°C for further analysis.

We measured eight body conformation traits in 104 GBG and 100 HBG, and these were: (1) Body height (BH): measured from the ground level to the highest point of the withers; (2) Body length (BL): measured from the top of the shoulder to the tip of the pin bone; (3) Chest depth (CD): the vertical distance from the highest point of the spine to the lowest point of the body at the level of the last rib; (4) Chest width (CW): measured between the inner surfaces of the chest at the level of the front legs; (5) Chest girth (CG): the circumference taken behind the shoulder blades in a vertical plane; (6) Rump width (RW): the horizontal distance between the most posterior points of the pin bones (ischial tuberosities); (7) Rump height (RH): measured from the ground level to the top of the pin bones; (8) Cannon circumference (CC): the horizontal circumference at the narrowest point of the left forelimb bone. The correlation heatmap and density trends of these traits were visualized using the R packages corrplot (ver. 0.94) and ggplot2 (ver. 3.4.4, https://ggplot2.tidyverse.org/).

### Genome sequencing and genotyping

Total DNA was extracted from the blood samples of 204 goats using the standard phenol-chloroform protocol. The concentration, integrity, and purity of the genomic DNA were assessed using agarose gel electrophoresis and a NanoDrop spectrophotometer (Thermo Fisher Scientific). During library construction, the genomic DNA was randomly broken into fragments of about 150 bp and after construction, the DNA library was sequenced by Compson Biotechnology Company using the DNBSEQ-T7 platform. The whole-genome sequencing raw data were preprocessed using the fastp tool (ver. 0.23.4, [22]) to obtain clean reads, and the clean data were further processed using Sentieon Genomics software (ver. 202308, [23]) for sequence alignment and variant detection. Briefly, the clean reads were aligned to the goat reference genome (ARS1.2, [24]) in bwa (ver. 0.7.17, [25]), and the aligned bam files were then sorted and duplicates marked to obtain a genomic variant call format (gVCF) file using the Sentieon Haplotyper module. All the gVCF files were subjected to joint variant calling using the Sentieon GVCFtyper module and the SNP variant type was split using the GATK (ver. 4.1.8.1, [26]) SelectVariants module. Quality control was performed in Plink v1.0.7 [27] to remove low-quality SNPs and individuals, including (i) SNPs located outside the autosomes; (ii) SNPs with missing genotypes >0.1; (iii) SNPs with minor allele frequencies <0.05; (iv) SNPs with a significant deviation (p< 0.001) from the Hardy–Weinberg equilibrium; and (v) individuals with a genotyping rate <90%.

### Population structure analysis

To guide the subsequent GWAS, we conducted principal component analysis (PCA), relatedness analysis, genetic distance analysis, and population genetic structure analysis. The PCA scores were calculated in Plink (ver. 0.76, [27], and their significance was evaluated with the EIGENSTRAT tool [28]. The kinship matrix (G matrix) was calculated in GEMMA (ver. 0.98.5, [29]), and the relatedness among individuals was visualized by using the R package heatmap (ver. 1.0.12). The hypothetical subpopulation (K) number was set to 1–8, and then Admixture (ver. 1.3.0, [30]) was used to calculate the cross-validation error (cv). The Python package PONG (ver. 1.5, [31]) was used to visualize the population structure.

### Genome-wide association studies

The GWAS of each body conformation trait was performed using GEMMA [29] and the first 10 PCs, gender, breed, and kinship matrix were included in the linear mixed model (LMM) as follows:


(1) Y=Wα+Xβ+Zu+e

Where *y* is the vector of the morphological trait; *W* is a matrix of covariates (including PCs, gender, and breed); *α* is the vector of corresponding coefficients including the intercept; *X* is the vector of SNP marker genotypes; *β* is the effect size of the SNP marker; *Z* is the loading matrix; *u* is the vector of random effects following a normal distribution *u*~N(0,*λτ^−1^K*), *K* is the kinship matrix derived from the SNP genotypes, *τ^−1^* is the variance of the residual effects, *λ* is the ratio between *τ^−1^* and *e*, and *e* is the residual error. To test the null hypothesis β = 0 for each SNP, the GEMMA performed the likelihood ratio and Wald tests. A significance threshold of p<8.33e-6 (1/12001271) was set to define a SNP as genome-wide significant. The Manhattan and quantile-quantile plots (QQ-plot) were drawn using the CMplot [32] and qqman [33] packages, respectively.

### Identification of candidate genes and enrichment analysis

After obtaining the signified SNPs and according to their physical positions on the ARS1.2 reference genome assembly, candidate genes were searched for within a ±200 kb region using the biomaRt tool [34]. LD between the SNP markers was calculated using the jar package Haploview (ver. 4.2, [35]). Finally, the Gene Ontology (GO) and Kyoto Encyclopedia of Genes and Genomes (KEGG) pathway analyses were performed using the Omicshare platform ( https://www.omicshare.com/tools/).

## RESULTS

### Descriptive statistics of phenotypic traits

**Figure 1:**
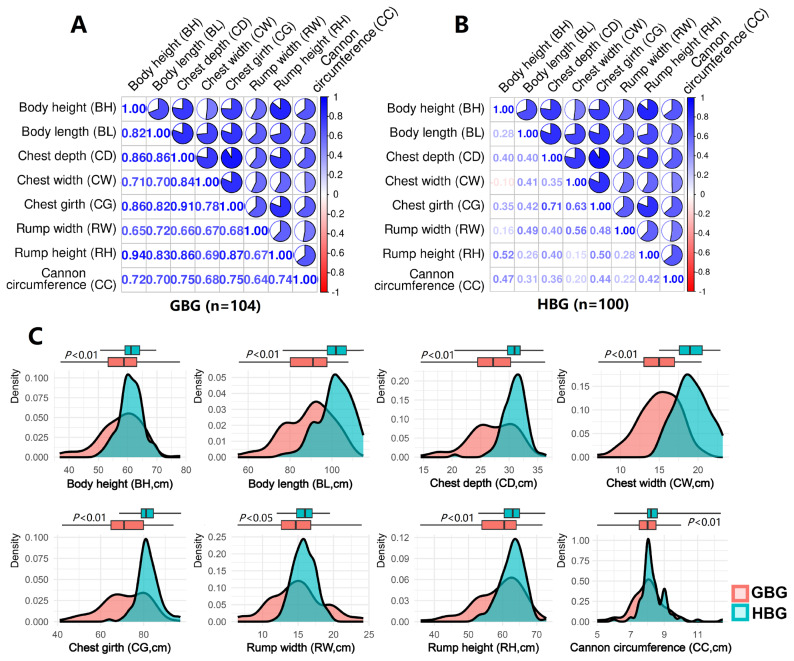
Goat breed and distribution characteristics of body conformation traits. (A, B) The heatmap of the correlation coefficient between any two of eight body conformation traits; (C) Comparison of the density trend line of eight body conformation traits between GBG and HBG. Box plot showing the maximum, Q1, mean, Q3, and minimum values. GBG, Guizhou black goat; HBG, Hezhang black goat.

Descriptive statistics of the eight body conformation traits of 204 adult goats (104 GBG and 100 HBG) showed a series of positive correlation coefficients among these eight traits (Figures 1A, 1B). In GBG, the highest correlation coefficient being between BH and RH (R = 0.94, p<0.01), and the smallest between RW and CC (R = 0.64, p<0.01). In HBG, the highest correlation coefficient being between CD and CG (R = 0.71, p<0.01), and the smallest between BH and CW (R = −0.1, p<0.01). Based on the density trend line and box plot (Figure 1C), the distribution characteristics between GBG and HBG were compared. The arithmetic mean and coefficients of variation (CV) of eight body conformation traits of GBG and HBG were calculated (Table 1). All body conformation traits including body height (BH: 57.7 and 61.0 cm), body length (BL: 89.0 and 101.6 cm), chest depth (CD: 27.1 and 30.9 cm), chest width (CW: 15.0 and 19.1 cm), chest girth (CG: 71.1 and 82.0 cm), rump width (RW: 14.9 and 15.9 cm), rump height (RH: 59.1 and 62.8 cm), cannon circumference (CC: 8.0 and 8.3 cm) were significantly different (p<0.01) between GBG and HBG. Overall, these two breeds were highly similar in body size, but HBG exhibiting a slight tendency towards larger dimensions.

### Genomic variants summary and population structure analysis

The whole genome re-sequencing of 204 goats produced 4,152.1 Gb of raw data, where each sequenced sample had approximately 136 million reads, and the Q20 (sequencing error rate<0.01), Q30 (sequencing error rate<0.001), and GC content values were 98.82%, 96.44%, and 42.3%, respectively. The average mapping rate showed 99% alignment with the goat reference genome, and the average sequencing depth was 7.5X. Following joint-calling on 204 goats, 36578352 SNPs were initially identified, which were reduced to 13954714 after quality control.

**Figure 2:**
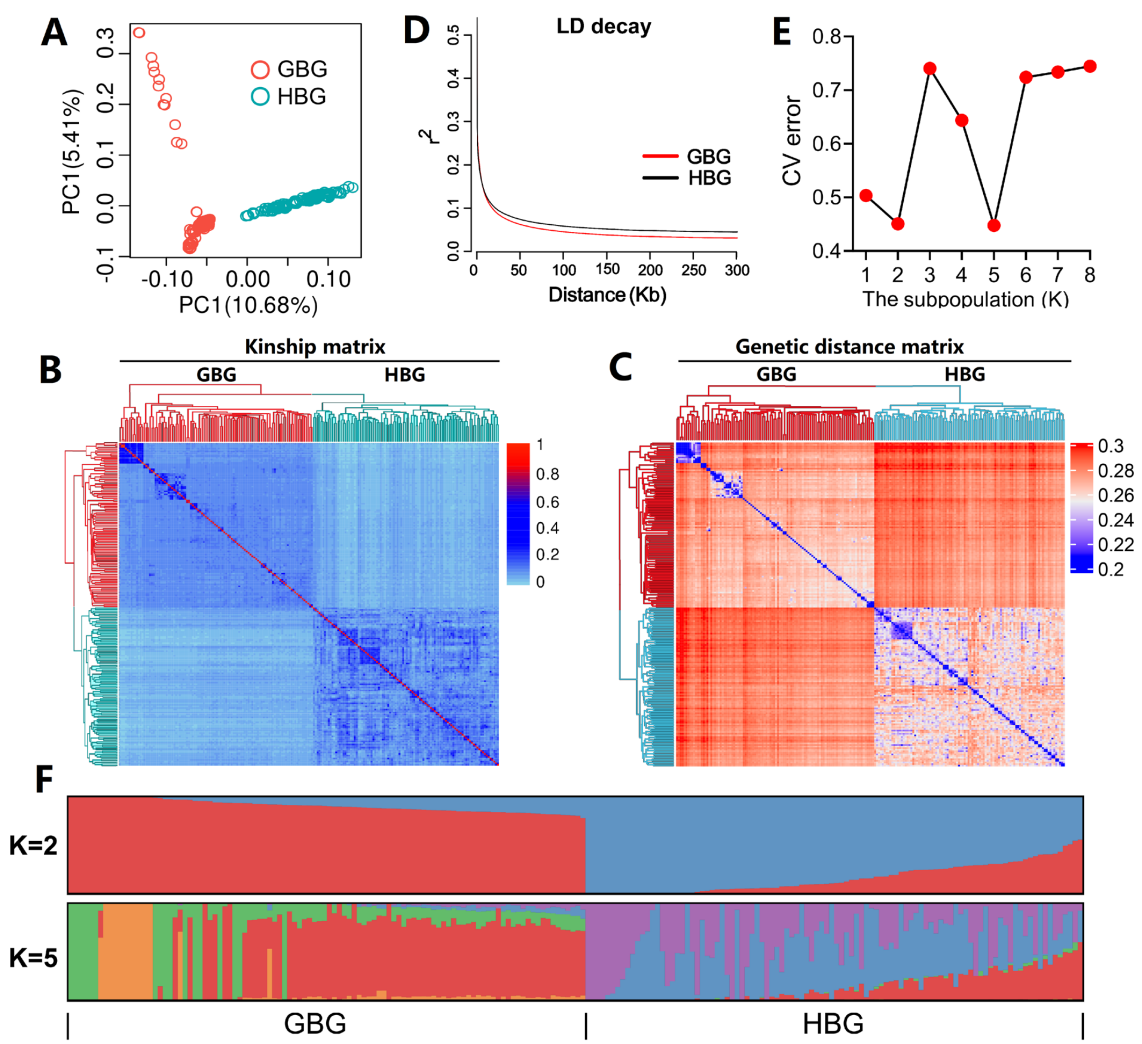
Population structure and kinship analyses based on whole genome SNPs. (A) A PCA score plot of all 204 goats based on whole genome sequence data. Top 10 PCs were included as covariates in the GWAS model due their statistical significance; (B) A heatmap of the kinship matrix of all 204 goats; (C) A heatmap of the genetics distance matrix for all 204 goats; (D) The linkage disequilibrium (LD) decay analysis for GBG and HBG; (E) The line chart of cross-validation error (CV) under different numbers of hypothetical subpopulations; (F) The barplot of the structure proportion when K = 2 and K = 5. Each vertical bar represents a sample. GBG, Guizhou black goats; HBG, Hezhang black goats; SNP, single nucleotide polymorphism.

The PCA results showed that PC1 and PC2 accounted for 10.68% and 5.41% of the total variance and that GBG and HBG were separated into two groups (Figure 2A). After performing the significance test, the top 10 PCs had statistical significance (p<8.8e-8, data not shown), suggesting that these PCs should be considered in the GWAS model. The heatmap of the relatedness coefficients showed that the two goat breeds were relatively independent (Figure 2B) and most relatedness coefficients were less than 0.1, suggesting very little genetic relatedness between the samples. Furthermore, the heatmap of the genetic distance matrix provided a clear delineation of breed independence, as shown in Figure 2C. The observed decay in LD demonstrated that GBG had a higher LD rate than HBG, implying a greater genetic diversity in the former and greater selective pressure (Figure 2D). The lowest two cross-validation error values (0.451 at K = 2, and 0.448 at K = 5) showed that the theoretical number of subpopulations was two and five (Figure 2E), which may be attributed to (1) the fact that two goat breeds were analysed, and (2) that five farms were included in the sample collection. Figure 2F illustrates the proportion of an individual’s genome derived from one of K ancestral populations, with the two ancestral origins corresponding most closely to each breed. These results showed the basic population structure and genetic relatedness within the two goat breeds, and suggested that PCA, breed factor, and kinship matrix should be included in the subsequent GWAS model.

### Genome-wide association study of six traits

**Figure 3:**
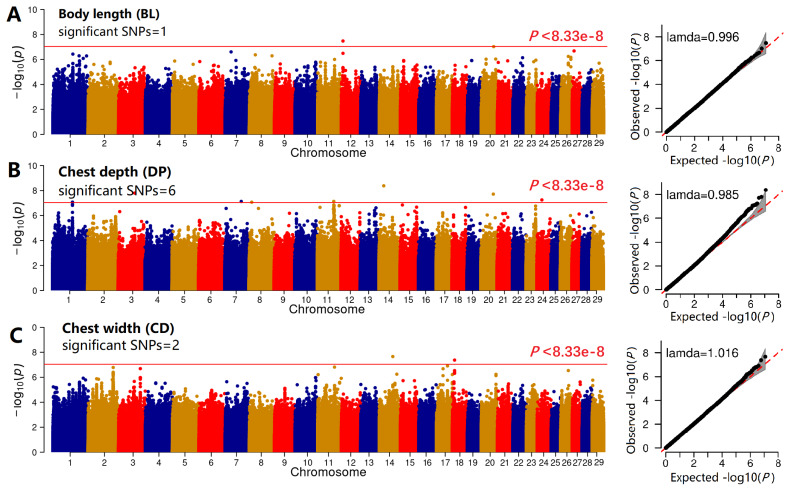
GWAS of three body conformation traits. The Manhattan and QQ plots of the GWAS results for body length (A), body depth (B), and chest width (C) of the Guizhou black goat and the Hezhang black goat. SNP, single nucleotide polymorphism; GWAS, genome-wide association studies.

**Figure 4:**
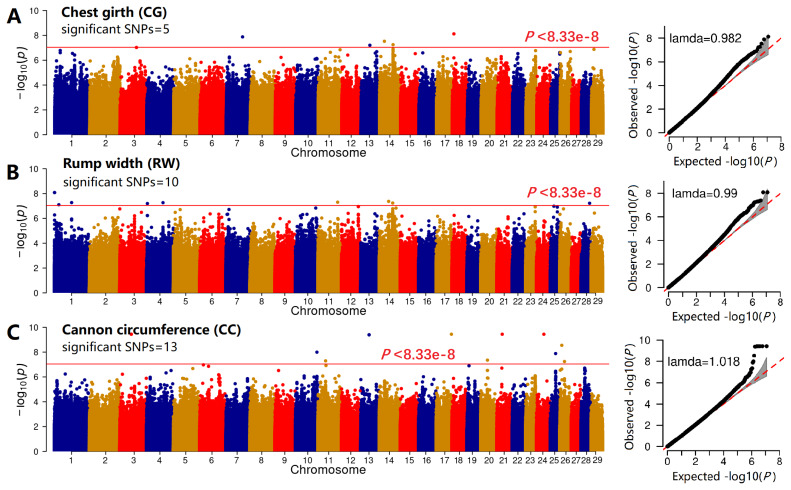
GWAS of three body conformation traits. The Manhattan and QQ plots of the GWAS results for chest girth (A), rump width (B), and cannon circumference (C) of the Guizhou black goat and the Hezhang black goat. SNP, single nucleotide polymorphism; GWAS, genome-wide association studies.

We further excluded SNPs on the sex chromosomes and removed the erroneous phenotypical records of two animals, ultimately retaining 202 goats with 12001271 SNPs for GWAS. The GWAS results were visualized as the Manhattan and QQ plots for BL, DP, and CD (Figures 3A–3C), the genomic inflation factors (BL, 0.996; DP, 0.985; CD, 1.016) indicated that the GWAS model was reliable. Here, only 1, 6, and 5 SNPs surpassed the threshold line, respectively. For CG, RW, and CC (Figures 4A–4C), the genomic inflation factors (BL, 0.982; DP, 0.990; CD, 1.018) indicated that the GWAS model was reliable and only 5, 10, and 13 SNPs surpassed the threshold line, respectively.

### Genome-wide association study of body height and rump height

**Figure 5:**
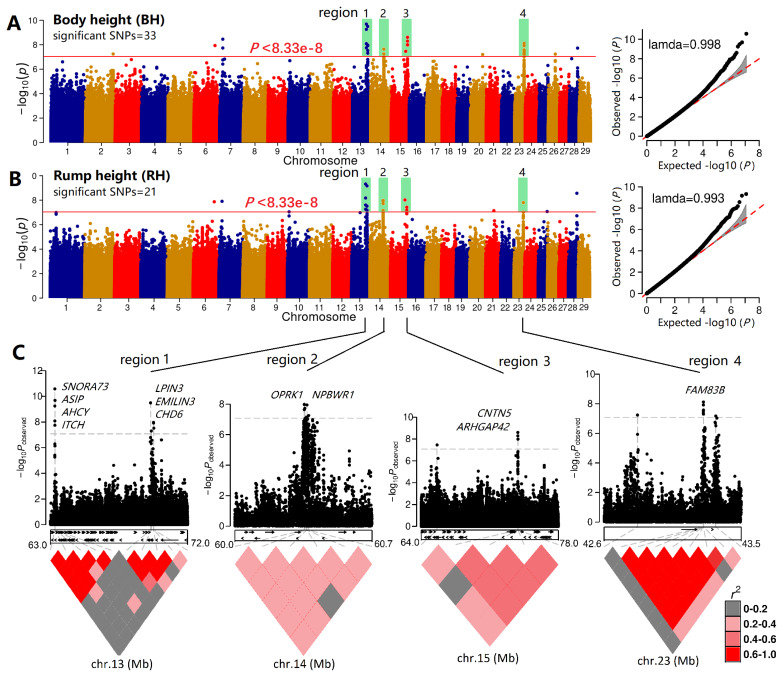
GWAS of body height and rump height. (A) The Manhattan and QQ plot of the GWAS of body height; (B) The Manhattan and QQ plot of the GWAS of Rump height; (C) The linkage disequilibrium and local Manhattan plot of four important regions significantly associated with body height and rump height in the Guizhou black goat and the Hezhang black goat. SNP, single nucleotide polymorphism; GWAS, genome-wide association studies.

The QQ plots of the GWAS for BH and RH showed normal lambda 0.998 and 0.993 values (Figures 5A, 5B), indicating that the GWAS results were reliable. The GWAS or BH and RH identified 33 and 21 significant SNPs, respectively, with the same significant peaks on chromosomes 13, 14, 15, and 23. These peak regions were chr13_63286230-69784740 (10 SNPs), chr14_60354209-60376549 (6 SNPs), chr15_ 65605417-73873841 (5 SNPs), and chr23_42819635-43332716 (9 SNPs) and Figure 5C shows the local Manhattan plot and local LD of these significant SNPs. Most or partial SNPs were tightly linked, mutually confirming their influence on the body conformation traits.

### Phenotypes changes in number of significant single nucleotide polymorphisms

**Figure 6:**
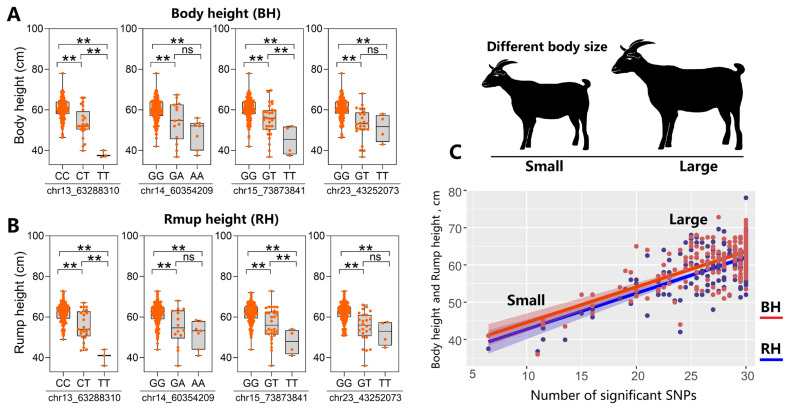
Effects of different genotypes on the body conformation traits. (A) The body height under different genotypes of four leading SNPs located in four important regions; (B) The Rump height under different genotypes of four leading SNPs located in four important regions of the Guizhou black goat and the Hezhang black goat; (C) The body height and rump height values changed with the number of significant SNPs. ** p<0.01. SNP, single nucleotide polymorphism.

The four leading SNPs were chr13_63288310, chr14_ 60354209, chr15_73873841, and chr23_43252073, respectively and the different genotypes of these four SNPs had various values of BH (Figure 6A) and RH (Figure 6B). For example, in the chr13_63288310 (C>T) locus, individuals with the CC, CT, and TT genotypes had varied body heights of 60.41, 53.63, and 38.10 cm, respectively. In the chr23_43252073 (G>T) locus, individuals with the GG, GT, and TT genotypes had rump heights of 62.17, 55.09, and 52.13 cm, respectively. When describing the trait value changes in numbers of significant SNPs (p<8.33e-8), results showed that individuals carrying more of these SNPs had higher BH and RH measurements (Figure 6C), illustrating the cumulative effects of these significant SNPs on body conformation traits.

### Single nucleotide polymorphism annotation and enrichment analysis

**Figure 7:**
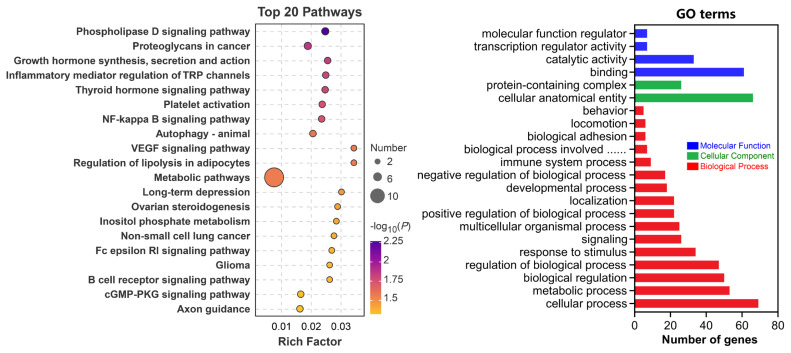
Function enrichment analysis of 102 annotated genes. (A) Bubble diagram of KEGG pathways; (B) Barplot of the GO enriched terms in the Guizhou black goat and the Hezhang black goat. KEGG, Kyoto Encyclopedia of Genes and Genomes; GO, Gene Ontology.

According to the physical locations of all 78 significant SNPs, a total of 102 genes within a 100 kb distance on either side were annotated. The majority of annotated genes were from chromosomes 13, 14, 15, and 23 (Table 2). The enrichment analyses, including KEGG pathways and GO terms, were performed based on all 102 genes. As shown in Figure 7A, the most significantly enriched KEGG pathways were the “Phospholipase D signaling pathway”, “Proteoglycans in cancer”, “Growth hormone synthesis, secretion and action”, “Inflammatory mediator regulation of TRP channels”, and “Thyroid hormone signaling”. As shown in Figure 7B, GO terms such as “cellular process”, “metabolic process”, and “biological regulation” belong to the Biological Process; the “cellular anatomical entity” and “protein-containing complex” belong to the Cellular Component; and the “binding”, “catalytic activity”, “transcription regulator activity” belongs to Molecular Function and were enriched.

## DISCUSSION

Given the rising global demand for goat meat driven by factors like population growth, dietary changes, and improved living standards, the genomic investigation into goats’ body conformation traits is very scientifically and economically valuable. This study performed a phenotypic and genetic analysis of eight body conformation traits on 204 goats using whole-genome sequencing. We identified millions of SNPs and finally found four regions located on chromosomes 13, 14, 15, and 23 that are strongly associated with body height and rump height.

Our population structure analysis revealed that GBG and HBG are genetically distinct with measurable introgression. Additionally, GBG showed greater genetic diversity than HBG, possibly due to less intensive selection pressure or broader environmental adaptation. These results were consistent with the geographic isolation and distinct breeding histories of the breeds. The HBG is sometimes considered to be a subtype of GBG, although it is a local breed in Guizhou Province, while GBG is a national breed representative of black goats in Guizhou Province [3]. The two breeds are similar in body shape except for the horns, where the GBG has normal-sized goat horns, but the HBG has no horns, also called a horse-headed goat. The hornlessness and bigger body size of HBG make it conducive to goat production, and it has good market competitiveness in Guizhou Province. To our knowledge, this is the first study to report and sequence genome data for HBG, providing valuable data for studies on environmental adaptation and production performance.

Recently, GBG received more research attention because of its high reproduction performance, resilience to coarse feed, and adaptability to adverse conditions. Zhao et al [36] identified the *KCNIP4*, *GFRA2*, and *DGKH* genes significantly associated with high reproductive performance, while Li et al [37] observed that the SNP polymorphisms in the *ACADM* gene were associated with intramuscular fat content. Previous research showed that the Guizhou black goat had a high level of genomic diversity and a low level of LD in the whole genome data [3] and based on the mRNAseq data from uterine, ovarian, and fallopian tube tissues, Zhou et al [38] identified the *AMH*, *GATA4*, and *DMRTA1* genes associated with reproduction traits of GBG. Interestingly, GBG is also an important grazing animal for evaluating toxic heavy metals, and Li et al explored their metabolomics profile in the liver [39] and blood [40] under cadmium exposure. Altogether, GBG is an excellent goat breed resource worthy of further research.

Body conformation traits in animals are complex quantitative characteristics regulated by a series of genes and biological pathways, also influenced by environmental factors. Therefore, there are several challenges to identifying the true variations that control these traits, including (1) measurement errors; (2) the polygenic model with small effects; and (3) significant influences from non-genetic factors, such as nutrition and climate. In the last 20 years, GWAS provided numerous compelling associations for complex traits and diseases in humans [41]. As a result, research in the field of animal genetics focusing on the mining of trait-related genes also benefited [42]. As far as we know, several other excellent studies have conducted GWAS on body conformation traits in goats, and Easa et al identified 241 SNPs and 238 candidate genes, including *CRADD, HMGA2*, and *MSRB3*, associated with body conformation traits in Russian Karachai goats [43]. Yang et al found two important regions (chr10:25988403-26102739 and chr11:88216493-89250659) and several candidate genes (such as *FNTB*, *CHURC1*, and *RNF144*) significantly associated with body conformation traits in Tashi goats [21]. Based on whole-genome sequencing and GWAS, Han et al identified 39 candidate genes (such as *ADIPOQ, CCDD39, PTPRT, ZNF215*, and *VRTN*) closely related to conformational traits in Zhongwei goats [44]. In the current study, a total of 78 significant and new SNPs and 102 newly annotated genes were identified for all eight body conformation traits. Interestingly, the SNP positions and candidate genes identified in these studies are not consistent, illustrating the complexity of these body conformation traits. It is therefore proposed that the implementation of a large-scale genomic analysis, such as a meta-GWAS, be conducted to identify significant SNPs that can commonly influence the body conformation traits of goats.

In other livestock, such as sheep, cattle, and pigs, the determinants of body shape resemble those of goats, and the regulating genes associated with these traits have been widely investigated. (1) In sheep, Liu et al [45] revealed 111 genes (including *ASAP1, CDK6, FRYL, NAV2, PTPRM, GPC6*, and *PTPRG*) in Tibetan sheep were associated with body conformation traits, while Tao et al [46] identified 39 genes (including *FOSL2, KCND2, TGFBI, LECT2*, and *TRAK1*) in Luzhong sheep, and Jiang et al [47] identified (including *KITLG*, *CADM2*, *MCTP1* and *COL4A6*) in Hu sheep. (2) In pigs, Deng et al. identified 60 significant genetic variants on chromosomes 7 and 13 [48], and Zhang et al. identified 82 SNPs on chromosomes 7, 10, 14, and 17 [49] that were associated with body length and body height. (3) In cattle, Liu et al [50] found genomic variants located on chromosomes 1, 3, and 7, and these annotated genes (including *CDH13, SIL1, CDC14A, TMRPSS15*, and *TRAPPC9*) were associated with body traits. Even when performing GWAS on as many as 1,314 Chinese Holstein cattle [51], the identified significant SNPs and candidate genes (including *DARC, GAS1, MTPN*, and *TMEM130*) were still different from those found in other studies, reflecting the diversity of the associated variants and annotated genes, akin to the diversity of species. This lack of effective common conclusions from studies implies that there is still much work to be done, both in terms of (1) sample size and (2) consideration of a multi-species integrative anlysis. Integrating comparative analyses across multiple species may offer new insights into the development of body size across different livestock, but it remains highly challenging.

In addition to the addition of 102 annotated genes, the main contribution of this study is the identification of four significantly variable genomic regions: chr13_63286230-69784740 (10 SNPs), chr14_60354209-60376549 (6 SNPs), chr15_65605417-73873841 (5 SNPs), and chr23_42819635-43332716 (9 SNPs). These significant SNPs were mutually verified for body height and rump height, possibly due to the high correlation of these two traits. Notably, the parameter “±100 Kb” used for gene annotation based on the SNP position was artificially defined and if the range of up- and down-steam distance were allowed to expand, the number of annotated genes obtained would be greater. Nevertheless, these SNP variants and genomic regions significantly associated with body height and rump height will be a valuable addition to the Goat Quantitative Trait Locus database ( https://www.animalgenome.org/cgi-bin/QTLdb/CH/index, [52]). These findings will also serve as an important reference for further investigations into the growth performance and body conformation traits of goats.

## CONCLUSION

In this study, eight body conformation traits were measured, and the GWAS was performed on 104 adult Guizhou black goats and 100 Hezhang black goats. All eight body conformation traits had a significantly positive correlation and were normally distributed. Based on the GWAS for each trait, 78 significant SNPs and 102 annotated genes were obtained, where body height and rump height had similar significant regions located in chr13_63286230-69784740 (10 SNPs), chr14_60354209-60376549 (6 SNPs), chr15_65605417-73873841 (5 SNPs), and chr23_42819635-43332716 (9 SNPs). All annotated genes were enriched in the KEGG pathway “Olfactory transduction” and GO terms including “cellular anatomical entity” and “molecular transducer activity”. These results have important implications for understanding body conformation and further molecular breeding in meat-type goats.

## Figures and Tables

**Table 1:** Descriptive statistics of body conformation traits

Body conformation traits	GBG (n = 104)				HBG (n = 100)			

	Mean	Max	Min	CV (%)	Mean	Max	Min	CV (%)
Body height (BH, cm)	57.7^A^	78.0	36.8	12.70	61.0^B^	69.6	50.4	6.30
Body length (BL, cm)	89.0^A^	108.0	55.0	12.80	101.6^B^	115.0	76.7	7.90
Chest depth (CD, cm)	27.1^A^	36.5	14.5	15.30	30.9^B^	36.0	20.5	6.70
Chest width (CW, cm)	15.0^A^	20.5	7.0	17.40	19.1^B^	23.2	15.0	10.90
Chest girth (CG, cm)	71.1^A^	94.0	41.0	14.90	82.0^B^	97.0	64.0	6.20
Rump width (RW, cm)	14.9^A^	24.2	6.5	24.70	15.9^B^	19.5	12.0	9.60
Rump height (RH, cm)	59.1^A^	72.0	36.0	11.60	62.8^B^	72.8	52.6	5.60
Cannon circumference (CC, cm)	8.0^A^	10.0	5.0	11.50	8.3^B^	12.5	6.0	9.50

A,B Means with Different capital letters differ when comparing GBG and HBG (p<0.01).

GBG, Guizhou black goat; HBG, Hezhang black goat; CV, coefficient of variation.

**Table 2:** Four important genomic regions associated with body height and rump height

Region No.	Interval length	SNP maker	Chr.	Position	PVE (%)	Reference homozygote	Heterozygote	Mutant homozygote	Annotated genes (±100 kb)
1	6.50 Mb	chr13_63286230	13	63286230	25.80	AA (83.8%)	AC (15.7%)	CC (0.5%)	*ZNF341, CHMP4B, RALY, SNORA73, ASIP, AHCY, ITCH, DYNLRB1, MAP1LC3A, PIGU, TP53INP2, NCOA6, GGT7, ACSS2, TOP1, U6, PLCG1, ZHX3, LPIN3, EMILIN3, CHD6*
		chr13_63288310	13	63288310	28.30	CC (86.3%)	CT (11.8%)	TT (1.5%)	
		chr13_63289494	13	63289494	21.40	CC (83.3%)	CT (14.2%)	TT (1.0%)	
		chr13_63291752	13	63291752	13.10	GG (85.3%)	GA (11.8%)	AA (1.0%)	
		chr13_63297458	13	63297458	23.20	AA (85.8%)	AG (12.8%)	GG (0.5%)	
		chr13_69573766	13	69573766	19.60	CC (90.2%)	CA (8.3%)	AA (1.0%)	
		chr13_69625659	13	69625659	14.20	CC (90.2%)	CT (8.3%)	TT (1.0%)	
		chr13_69764797	13	69764797	18.10	CC (90.2%)	CT (9.3%)	TT (0.5%)	
		chr13_69770755	13	69770755	17.20	TT (89.7%)	TC (6.9%)	CC (2.5%)	
		chr13_69784740	13	69784740	11.20	CC (85.8%)	CA (9.3%)	AA (4.9%)	
2	22 Kb	chr14_60354209	14	60354209	17.00	GG (86.8%)	GA (6.9%)	AA (3.4%)	*ATP6V1H, OPRK1, NPBWR1, RB1CC1, ST18*
		chr14_60357203	14	60357203	15.10	TT (87.3%)	TG (8.3%)	GG (2.9%)	
		chr14_60365649	14	60365649	18.10	AA (87.8%)	AG (6.4%)	GG (3.9%)	
		chr14_60368174	14	60368174	12.10	TT (85.8%)	TC (5.4%)	CC (4.9%)	
		chr14_60368200	14	60368200	9.70	GG (85.8%)	GA (4.4%)	AA (4.9%)	
		chr14_60376549	14	60376549	13.70	CC (88.7%)	CT (5.9%)	TT (3.9%)	
3	8.27 Mb	chr15_65605417	15	65605417	17.80	TT (84.3%)	TC (11.3%)	CC (2.0%)	*ALKBH8, ELMOD1, U6, CNTN5, ARHGAP42*
		chr15_73859174	15	73859174	18.70	TT (84.8%)	TC (12.3%)	CC (2.5%)	
		chr15_73862260	15	73862260	20.70	GG (84.3%)	GA (13.2%)	AA (1.5%)	
		chr15_73873816	15	73873816	20.60	GG (83.3%)	GC (14.2%)	CC (2.0%)	
		chr15_73873841	15	73873841	21.30	GG (82.8%)	GT (14.7%)	TT (2.0%)	
4	0.51 Mb	chr23_42819635	23	42819635	10.60	TT (82.8%)	TC (13.2%)	CC (2.5%)	*TINAG, HCRTR2, GFRAL, HMGCLL1, FAM83B*
		chr23_43251687	23	43251687	19.90	TT (79.9%)	TA (18.1%)	AA (2.0%)	
		chr23_43252073	23	43252073	10.20	TT (83.3%)	TC (12.3%)	CC (2.0%)	
		chr23_43252140	23	43252140	11.60	TT (82.4%)	TA (13.2%)	AA (2.5%)	
		chr23_43252197	23	43252197	13.10	GG (80.9%)	GA (16.2%)	AA (2.0%)	
		chr23_43252276	23	43252276	22.10	CC (79.4%)	CG (17.2%)	GG (2.5%)	
		chr23_43252399	23	43252399	20.30	TT (80.4%)	TC (15.2%)	CC (3.9%)	
		chr23_43252532	23	43252532	19.30	GG (81.4%)	GC (15.7%)	CC (2.0%)	
		chr23_43332716	23	43332716	14.50	CC (78.4%)	CA (14.7%)	AA (4.9%)	

PVE refers to the percentage of the phenotypic variation explained.

If the sum of the proportions of Reference Homozygote, Heterozygote, and Mutant Homozygote was less than 1, it indicates that there are some individuals whose genotypes were missing.

SNP, single nucleotide polymorphism.
